# Neutrophil-to-lymphocyte ratio predicts early mortality in females with metastatic triple-negative breast cancer

**DOI:** 10.1371/journal.pone.0243447

**Published:** 2020-12-07

**Authors:** Gabriel de la Cruz-Ku, Diego Chambergo-Michilot, J. Smith Torres-Roman, Pamela Rebaza, Joseph Pinto, Jhajaira Araujo, Zaida Morante, Daniel Enriquez, Claudio Flores, Renato Luque, Antonella Saavedra, Maria Lujan, Henry Gomez, Bryan Valcarcel

**Affiliations:** 1 Facultad de Ciencias de la Salud, Escuela de Medicina Humana, Universidad Científica del Sur, Lima, Perú; 2 Unidad de Investigación Básica y Translacional, Oncosalud-AUNA, Lima, Perú; 3 Department of Surgery, Mayo Clinic, Rochester, MN, United States of America; 4 Latin American Network for Cancer Research (LAN-CANCER), Lima, Peru; 5 Department of Cardiology Research, Torres de Salud National Research Center, Lima, Peru; 6 Department of Breast Surgical Oncology, National Institute of Neoplastic Diseases, Lima, Peru; 7 Escuela de Medicina Humana, Universidad Privada San Juan Bautista, Lima, Perú; 8 Department of Hematology and Medical Oncology, National Institute of Neoplastic Diseases, Lima, Peru; American Society for Investigative Pathology, UNITED STATES

## Abstract

**Background:**

The aim of this study was to determine the utility of the neutrophil-to-lymphocyte ratio (NLR) as a biomarker for predicting early-mortality (<2 years) among females with metastatic triple-negative breast cancer (mTNBC).

**Methods:**

We reviewed 118 medical records of females with mTNBC. The cut-off value for the NLR (<2.5 and ≥2.5) was determined with receiver operating characteristic curves (area under the curve: 0.73; 95% CI: 0.62–0.85). Survival curves were estimated using the Kaplan-Meier method and compared with the Log-rank test. Multivariate Cox regression was used to identify the risk of mortality at two years. Moreover, we performed sensitivity analyses with different cut-off values and a subgroup analysis in females that only received chemotherapy.

**Results:**

The median follow-up was 24 months. Females with NLR ≥2.5 had a poor overall survival compared to females with NLR <2.5 (6% vs. 28%, p<0.001) at two years. This outcome remained when we stratified for females that only received chemotherapy (8% vs. 36%, p = 0.001). Multivariate analyses identified NLR ≥2.5 as a poor prognostic risk factor for mortality in the entire population (HR: 2.12, 95% CI: 1.32–3.39) and among females that received chemotherapy (HR: 2.68, 95% CI: 1.46–4.92).

**Conclusion:**

The NLR is an accessible and reliable biomarker that predicts early mortality among females with mTNBC. Our results suggest that females with high NLR values have poor prognosis despite receiving standard chemotherapy. Health providers should evaluate the possibility to enroll these patients in novel immunotherapy trials.

## Introduction

Triple-negative breast cancer (TNBC) is a heterogeneous and aggressive subtype of breast cancer. It is defined as a lack of expression of estrogen and progesterone hormone receptors and human epidermal growth factor receptor 2 (HER2) and has a high lethality and limited therapeutic options [1]. In contrast to other subtypes, females with TNBC have worse survival outcomes and the only available systemic treatment is chemotherapy [1, 2]. Despite these poor outcomes and restricted treatment alternatives, few authors have studied effective biomarkers to predict survival prognosis in Latin American countries [3–6].

It is known that the immune system plays an important role in the pathophysiology of neoplasms. Studies have reported that high concentrations of blood neutrophils are associated with poor survival in many cancers [7, 8]. However, other studies identified favorable survival outcomes in females with a high concentration of lymphocytes in breast cancer [9, 10]. For this reason, the neutrophil-to-lymphocyte ratio (NLR) has demonstrated to be a useful biomarker to predict survival outcomes in breast cancer [11–13].

Most of the research in this field has been carried out in Eastern or Western countries [14, 15]. A meta-analysis in all breast cancer subtypes and tumor stages found that the NLR only predicts mortality in TNBC and HER2 subtypes; however, the authors report a high heterogeneity among the studies [15]. In addition, a meta-analysis of three studies, including American females with early-stage breast cancer, reported that the NLR did not predict survival outcomes, irrespective of the subtypes [14].

These studies suggested the need to perform more research regarding the prognostic value of NLR to decrease the heterogeneity and determine the utility of NLR in different ethnicities or geographical areas [14, 15]. Most of the single studies that studied the prognostic value of this biomarker among females with metastatic TNBC (mTNBC) analyzed all breast cancer subtypes [16–18], and reports that focused on TNBC excluded patients with a metastatic stage at diagnosis [19]. Indeed, few studies have explored the usefulness of the NLR in females with mTNBC. For example, a small retrospective cohort of 57 females with mTNBC found that NLR ≥2.5 predicts poor progression-free survival [20]. Based on these previous studies, we aimed to determine the utility of the NLR as a biomarker for predicting early-mortality (<2 years) among females with mTNBC.

## Material and methods

### Study design and population

We reviewed the medical records of females with mTNBC, diagnosed and treated at the National Institute of Neoplastic Diseases in Lima, Peru between January 2000 and November 2017. Clinical records with the code C50 –“Malignant neoplasm of breast” of the International Statistical Classification of Diseases-10^th^ edition [21] were identified in the database of the Department of Medical Oncology and selected for analysis. We included females with metastasis at breast cancer diagnosis confirmed by computed tomography scan or magnetic resonance imaging and with complete data of receptor status in the immunohistochemistry report.

### Variables and management

Demographic, clinical, and pathological variables were recorded at breast cancer diagnosis. We used the Charlson comorbidity index (CCI) to classify the comorbidities of the women included in the study. The CCI is an instrument that evaluates the presence of 19 medical conditions and assigns them with a score from zero to six based on their impact on survival outcomes. However, the CCI does not include hypertension. Hence, we computed a hypertension-augmented CCI (hCCI) and assigned a weight of “one” to females with this condition, as in a previous study [22]. Tumor size and lymph node status were classified according to the 7^th^ edition of the American Joint Committee on Cancer [23]. Patients were treated with chemotherapy regimens according to the National Comprehensive Cancer Network (NCCN) guideline on Breast Cancer (Version 3.2018) [2]. The complete list of chemotherapy agents and regimes are shown in S1 Appendix.

### Exposure definition

The results of the peripheral blood count at diagnosis were used to calculate the NLR by dividing the absolute neutrophil count by the absolute lymphocyte count. We used receiver operating characteristic (ROC) curves to calculate the area under the curve (AUC). Cut-off values were selected with the sensitivity equals the specificity method (NLR = 2.5; AUC: 0.73; 95% confidence interval [CI]: 0.62, 0.85) to obtain a balance estimate between the probability of prognosing mortality and that of predicting survival (S2 Appendix). We performed a sensitivity analysis with two different cut-off points, identified with the Youden index (NLR = 3) and maximization of specificity (NLR = 7) methods (S3 and S4 Appendix).

### Data analysis and final outcomes

We described the clinicopathological and treatment characteristics of the entire population and according to the NLR group (<2.5 vs. ≥2.5). For descriptive analysis, age was grouped into three categories according to the percentiles of the population (<25^th^, 25^th^-75^th^, and >75^th^). Categorical variables were compared with the Chi-square test. Overall survival (OS) was defined as the time between mTNBC diagnosis and mortality by any cause or the end of the study (November 2017). Due to the high mortality rate in mTNBC, females were followed for two years. Two crucial variables had missing values (tumor size and lymph node status). We used the Chi-squared test to determine whether the missing variables in the exposure variable were related to other variables in the database, using the “finalfit” package. However, we were unable to identify a clear missing pattern. Hence, we assumed a “missing completely at random” condition and used the listwise deletion technique to handle the missing variables.

Survival probabilities between the two groups were estimated with the Kaplan-Meier method and compared with the Log-rank test. We fitted a multivariate Cox proportional hazard regression model to estimate the risk of mortality between both NLR groups in the overall population, adjusting for age, hCCI, tumor size, lymph node status, number of sites of metastases, and use of chemotherapy. Our model included variables that are typically related to cancer mortality and variables that were associated with mortality in the univariate analysis of our dataset. Furthermore, we performed a subgroup analysis in females that only received chemotherapy (n = 77, 65.3%) and estimated survival probabilities and mortality risk between NLR groups using the abovementioned methods. We reported our outcomes with adjusted hazard ratios (HR) and 95% confidence intervals (CI). Results with a p-value <0.05 were considered statistically significant. We used the R version 4.0.2 software for the statistical analyses.

### Ethics statement

The study was approved by the institutional review board of the National Institute of Neoplastic Diseases’ (study protocol code: INEN 16–46), which waived the need for inform consent. Therefore, personally identifiable information of the participants was anonymized upon extraction of the relevant data for the study, and patients were coded using numbers (e.g., 1, 2, or 3, and so on).

## Results

Of the 2,007 females with TNBC, 131 had mTNBC. Thirteen females were excluded because their medical records had missing values in the exposure variable, leaving 118 females with mTNBC for this analysis. At diagnosis, most females were between 41 and 59 years of age, were postmenopausal, had a hCCI score of 6, a T4 clinical tumor size status, an N1 clinical lymph node status, and a histologic grade III (Table 1). Ductal carcinoma (87.3%) and invasive lobular carcinoma (3.4%) were the most common types of breast cancer. There were 12 different sites of metastases, with a total of 205 metastases. Of these, the most frequent site was the lungs (63, 30.7%), followed by the bones (n = 47, 22.9%), the liver (n = 37, 18%), and the brain (n = 22, 10.7%).

**Table 1:** Clinicopathological and treatment characteristics of females with mTNBC.

Characteristics	No. of females (n = 118)	Percentage (%)
Group age in years		
≤40	32	27.1
41–59	56	47.5
≥60	30	25.4
Menopausal status		
Premenopausal	43	36.4
Postmenopausal	72	61.0
Missing	3	2.5
hCCI^a^		
Score 6	91	77.1
Score ≥7	27	22.9
Clinical tumor size		
T0	3	2.5
T2	13	11.0
T3	14	11.9
T4	85	72.0
Missing	3	2.5
Clinical lymph node status		
N0	14	11.9
N1	48	40.7
N2	25	21.2
N3	27	22.9
Missing	4	3.4
Histologic grade		
Grade II	11	9.3
Grade III	71	60.2
Missing	36	30.5
Site of metastases		
1 organ	58	49.2
2 organs	36	30.5
3 organs	14	11.9
4 organs	9	7.6
5 organs	1	0.8
Chemotherapy	77	65.3

^a^hCCI, hypertension-augmented Charlson comorbidity index

Table 2 shows a similar distribution of the clinicopathological and treatment characteristics of the females according to the NLR. Regarding the clinical variables at diagnosis, cases with an NLR ≥ 2.5 were older, had a higher hCCI, and higher clinical lymph node staging, although without statistical significance. On the contrary, females with a low NLR status had a higher histologic grade and received chemotherapy more frequently; being results with no statistical significance.

**Table 2:** Clinicopathological and treatment characteristics according to NLR status.

Characteristics	NLR^a^ <2.5	NLR^a^ ≥2.5	P-value
No. of females	39	79	
Group age in years			0.651
≤40	12 (30.8)	20 (25.3)	
41–59	19 (48.7)	37 (46.8)	
≥60	8 (20.5)	22 (27.8)	
Menopausal status			0.949
Premenopausal	15 (38.5)	28 (35.4)	
Postmenopausal	23 (59.0)	49 (62.0)	
Missing	1 (2.6)	2 (2.5)	
hCCI^b^			0.507
Score 6	32 (82.1)	59 (74.7)	
Score ≥7	7 (17.9)	20 (25.3)	
Clinical tumor size			0.297
T0	0 (0.0)	3 (3.8)	
T2	6 (15.4)	7 (8.9)	
T3	2 (5.1)	12 (15.2)	
T4	30 (76.9)	55 (69.6)	
Missing	1 (2.6)	2 (2.5)	
Clinical lymph node status			0.906
N0	5 (12.8)	9 (11.4)	
N1	18 (46.2)	30 (38.0)	
N2	7 (17.9)	18 (22.8)	
N3	8 (20.5)	19 (24.1)	
Missing	1 (2.6)	3 (3.8)	
Histologic grade			0.374
Grade II	5 (12.8)	6 (7.6)	
Grade III	25 (64.1)	46 (58.2)	
Missing	9 (23.1)	27 (34.2)	
Site of metastases			0.768
1 organ	22 (56.4)	36 (45.6)	
2 organs	11 (28.2)	25 (31.6)	
3 organs	4 (10.3)	10 (12.7)	
4 organs	2 (5.1)	7 (8.9)	
5 organs	0 (0.0)	1 (1.3)	
Chemotherapy	28 (71.8)	49 (62.0)	0.399

^a^Neutrophil-to-lymphocyte ratio

^b^hCCI, hypertension-augmented Charlson comorbidity index

**Fig 1:**
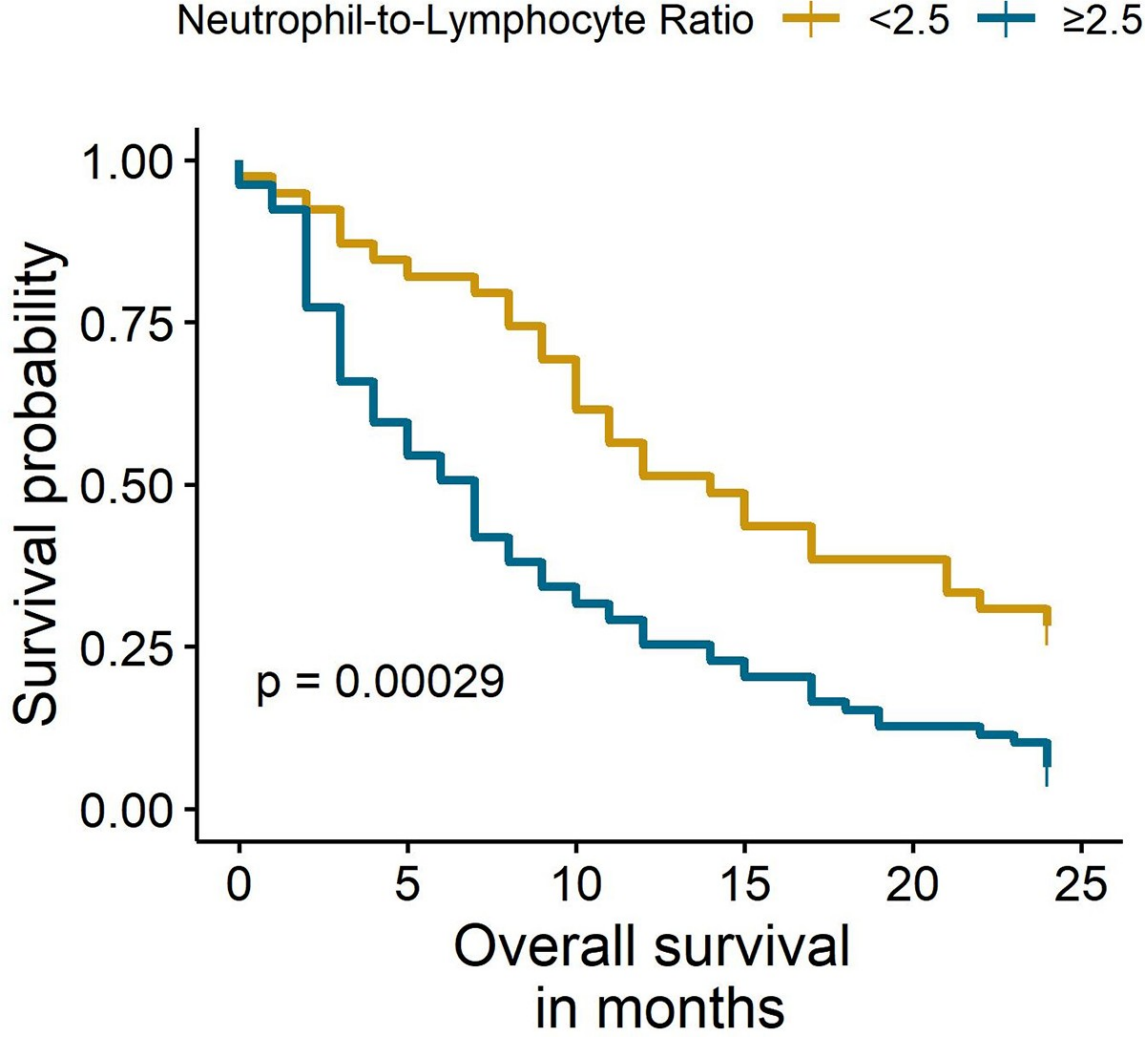
Overall survival of total females according to NLR status.

**Fig 2:**
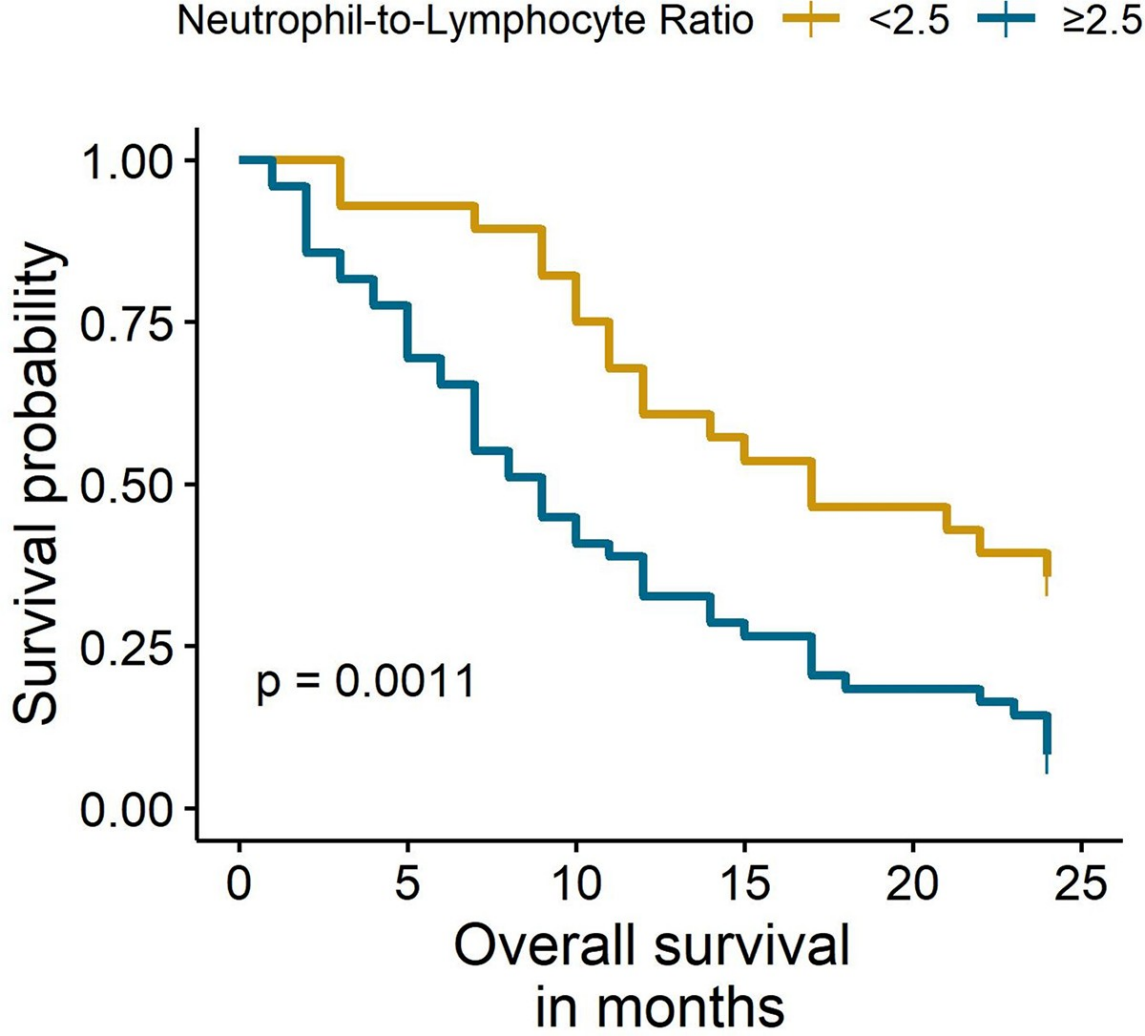
Overall survival of females with chemotherapy according to NLR status.

The median follow-up was 24 months. The Kaplan-Meier analysis showed significant differences in OS between females with NLR <2.5 and NLR ≥2.5 (6% vs. 28%, p < 0.001) at two years (Fig 1). Similarly, the subgroup analysis of females that only received chemotherapy identified a worse OS in females with an NLR ≥ 2.5 (8% vs. 36%, p = 0.001) (Fig 2). Multivariate Cox regression analyses found that an NLR ≥ 2.5 was an independent prognostic factor for mortality in the entire population (HR: 2.12, 95% CI: 1.32–3.39) and in females that only received chemotherapy (HR: 2.68, 95% CI: 1.46–4.92) (Table 3). The sensitivity analyses with different cut-off values (NLR = 3 and NLR = 7) showed similar results (S5 Appendix).

**Table 3:** Multivariate Cox regression analysis of mortality factors in the entire cohort and in females that only received chemotherapy.

Characteristics	OS in the entire cohort			OS in patients only receiving chemotherapy		
	HR	95% CI	P-value	HR	95% CI	P-value
NLR^a^						
< 2.5	Ref	-	-	Ref	-	-
≥ 2.5	2.12	1.32–3.39	0.002	2.68	1.46–4.92	0.001
Age	1.00	0.98–1.01	0.748	1.00	0.98–1.03	0.821
hCCI^b^						
Score 6	Ref	-	-	Ref	-	-
Score ≥ 7	0.77	0.45–1.32	0.342	0.65	0.30–1.41	0.281
Tumor size						
T0-3	1	-	-	Ref	-	-
T4	1.15	0.71–1.85	0.577	0.72	0.37–1.39	0.325
Lymph node status						
N0-1	Ref	-	-	Ref	-	-
N2-3	1.07	0.70–1.64	0.758	1.35	0.75–2.43	0.319
Site of metastases						
1 organ	Ref	-	-	Ref	-	-
≥ 2 organs	1.17	0.77–1.77	0.473	0.97	0.56–1.68	0.912
Chemotherapy						
No	Ref	-	-	-	-	-
Yes	0.41	0.26–0.64	<0.001	-	-	-

^a^Neutrophil-to-lymphocyte ratio

^b^hCCI, hypertension-augmented Charlson comorbidity index

## Discussion

This study shows that the NLR is a reliable biomarker for predicting poor OS in females with mTNBC. Previous studies analyzed all types of breast cancer or reported regression models without adjusting for tumor stage when TNBC was analyzed separately, primarily in Western and Eastern populations [14, 15]. Moreover, a single study on mTNBC reported a significant association between a high NLR and poor progression-free survival [20]. Other reports have focused on studying this biomarker in patients with early-stage TNBC. One study found that an NLR > 3 before surgery was a prognostic factor for a poor OS in TNBC females with stage I-IIIA [24], and two other studies that included non-metastatic TNBC cases supported the previous statement [25, 26]. This association was further confirmed in a recent meta-analysis that identified a high NLR as a factor for poor OS in females with unspecific breast cancer (HR: 1.78) and TNBC (HR: 2.18) [14]. We built on these experiences and identified the usefulness of the NLR to predict mortality using three distinct cut-off values in a cohort of Peruvian women with mTNBC.

The prediction of survival using the NLR in females with metastatic breast cancer has shown conflicting results. Takuwa *et al*. reported a worse OS in females with an NLR ≥ 1.90 [27], while Vernieri *et al*. found the same outcome with an NLR ≥ 2.5 [20]. In contrast, one study found a correlation between NLR and OS in the univariate analysis, but a non-significant association in multivariate Cox regression analysis [18]. The researchers argued that the survival rate with this biomarker depends on the tumor stage at diagnosis, performance status according to the Eastern Cooperative Oncology Group (ECOG) scale, and the location of the metastasis. Few studies have considered the effect of the ECOG scale on the regression model. Although this variable predicts poor survival outcomes in women with metastatic breast cancer [28, 29], Kumar *et al*. [30] identified that the NLR was a prognostic factor of OS independently of the ECOG score in a large cohort of females with oncological disease (15% with breast cancer).

Our study presents several differences compared to the study by Rubio *et al*. [18], which analyzed all subtypes of breast cancer, 14.5% of which were TNBC. We limited our population to stage IV TNBC. Then, we only focused on females with metastases at diagnosis, while in the study by Rubio *et al*. the prevalence of metastatic breast cancer at diagnosis was 44.5%. In addition, we adjusted our model to hCCI, tumor size, and lymph node status. Therefore, these differences may explain the significant association between NLR in our multivariate analysis and the analysis made by Rubio *et al*. Moreover, these findings may also suggest that each subtype of metastatic breast cancer has a different prognostic profile.

Two meta-analyses identified that most studies used ROC curves to determine the cut-off values of the NLR (range: 2–4) in breast cancer [19, 31]. Similar to these reports, we employed the sensitivity equals specificity method to provide a useful cut-off point in clinical practice. This method balances the trade-off between sensitivity and specificity to adequately identify people with a probability of dying, avoiding a high rate of false positives. Moreover, we employed two other methods in the sensitivity analysis and identified the same outcomes. Therefore, with the Youden index, we corroborated the effectiveness of the biomarker, and the maximization of specificity method provided a useful cut-off point to achieve a highly reliable prediction of mortality in women with mTNBC, minimizing the rate of false positives (i.e., to provide a threshold to aid clinicians in predicting mortality when a patient have a high NLR).

We further made a subgroup analysis including females that only received chemotherapy to address the importance of the NLR in this population. Our results remained robust in the sub-population analysis, supporting the premise that the NLR is a useful biomarker to predict survival in mTNBC. Moreover, this outcome suggests that mTNBC patients with NLR ≥2.5 have an increased risk of mortality despite receiving standard chemotherapy regimens. Therefore, it would be relevant to evaluate new treatment approaches, as well as close follow-up for patients with high NLR values, particularly in immunotherapy trials. In addition, we did not exclude females with systemic comorbidities; instead, we controlled this factor with the hCCI in the multivariate analysis, resembling the daily clinical practice and making our outcomes useful in this context.

This study has some limitations. We excluded medical records in the regression analysis due to missing data. However, our diagnostic analysis of the missing variables suggested that listwise deletion was the appropriate method to use. Although we did not include the performance status, the hCCI was used to address the distribution of background comorbidities in the females. Due to the low number of females that did not receive chemotherapy, we could not perform further analysis in this subgroup. Given that mTNBC is an aggressive disease, most patients died within two years of observation, impeding conventional estimations up to five years. We assessed females from a single center, and thus, extrapolation of our results should be made with caution. Besides, we cannot extrapolate it for other Latin American populations, so we encourage future research about the comparison of TNBC mortality between these populations. Finally, our analysis also provides the performance of this biomarker with different cut-off values and its utility in females under chemotherapy.

## Conclusion

In a cohort of Peruvian women with mTNBC, the present study showed that NLR is a useful predictor of poor OS. Values of NLR ≥2.5 were associated with mortality in the main analysis and in the subgroup of patients that received chemotherapy. Our results suggest that standard chemotherapy is not beneficial for this high-risk population. Therefore, health providers should evaluate the possibility to enroll these patients in novel immunotherapy trials with close follow-up.

## Supporting information

S1 Appendix Complete list of chemotherapy agents and regimes. (DOCX)

S2 Appendix ROC curve of the NLR biomarker with the sensitivity equals the specificity method. (PDF)

S3 Appendix ROC curve of the NLR biomarker with the Youden index–o–Youden statistic. (PDF)

S4 Appendix ROC curve of the NLR biomarker with the maximization of specificity method. (PDF)

S5 Appendix Multivariate analysis of different cut-off values of the NLR. (DOCX)

S1 Data (XLSX)
