# Supplementary material for: Neutrophil-to-lymphocyte ratio predicts early mortality in females with metastatic triple-negative breast cancer
Source: PLoS One. 2020 Dec 7;15(12):e0243447. doi: 10.1371/journal.pone.0243447 (PMC7721150; doi:10.1371/journal.pone.0243447)
Supplement: S1 Appendix — (DOCX) [file pone.0243447.s001.docx]

S1 Appendix. Treatment list

| Based regimens | Number of patients |
| --- | --- |
| Taxanes + Anthracyclines | 29 |
| Anthracyclines | 18 |
| Taxanes | 14 |
| Anthracyclines + Taxanes + Platinum based | 10 |
| Platinum based | 3 |
| Antimetabolites | 2 |
| Taxanes + Platinum based | 1 |
