# Supplementary figures and images for: Neutrophil-to-lymphocyte ratio predicts early mortality in females with metastatic triple-negative breast cancer
Source: PLoS One. 2020 Dec 7;15(12):e0243447. doi: 10.1371/journal.pone.0243447 (PMC7721150; doi:10.1371/journal.pone.0243447)

ROC Curve. Criterion: SpEqualSe

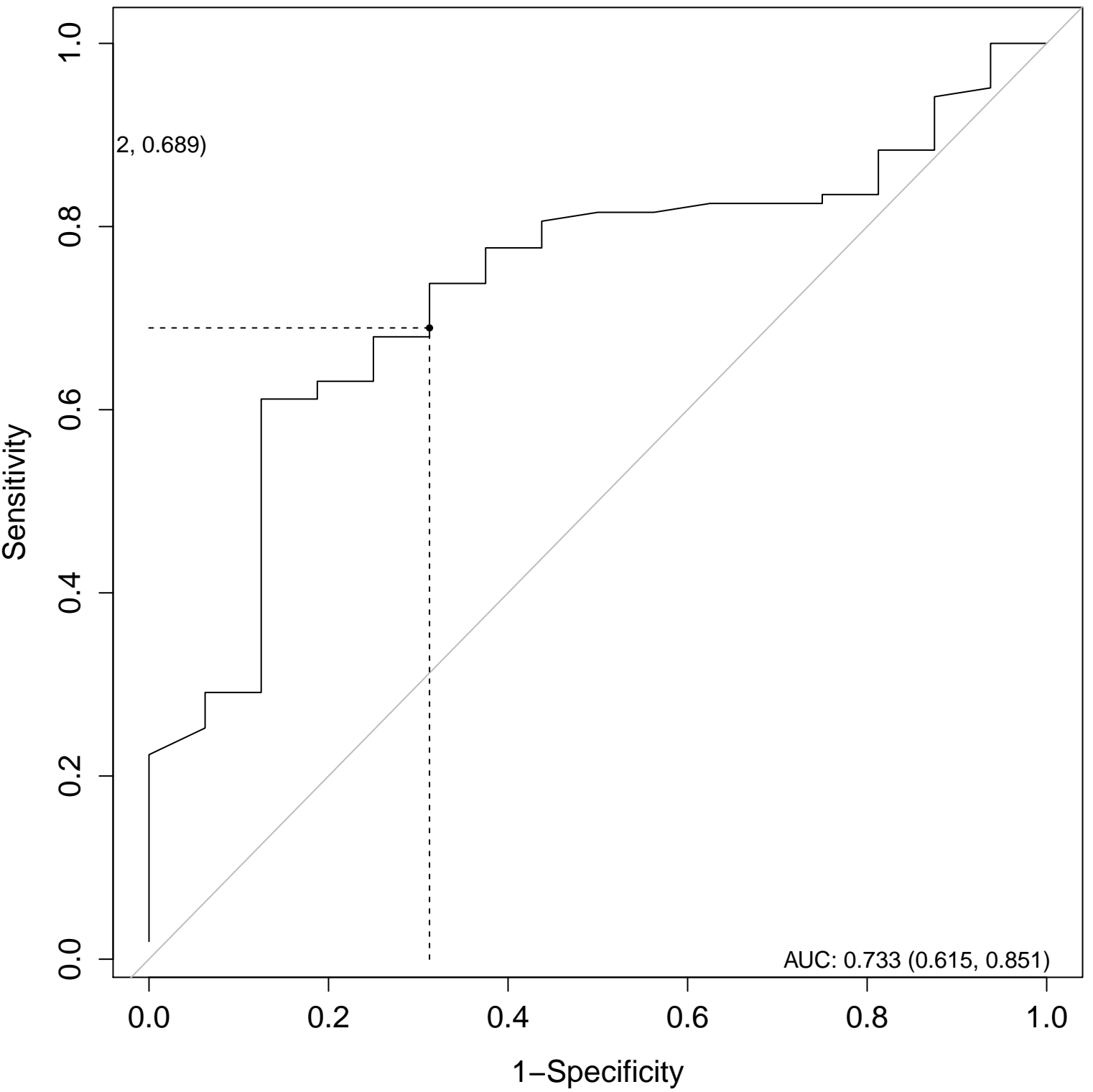

Supplement: S2 Appendix — (PDF) [file pone.0243447.s002.pdf]

ROC Curve. Criterion: Youden

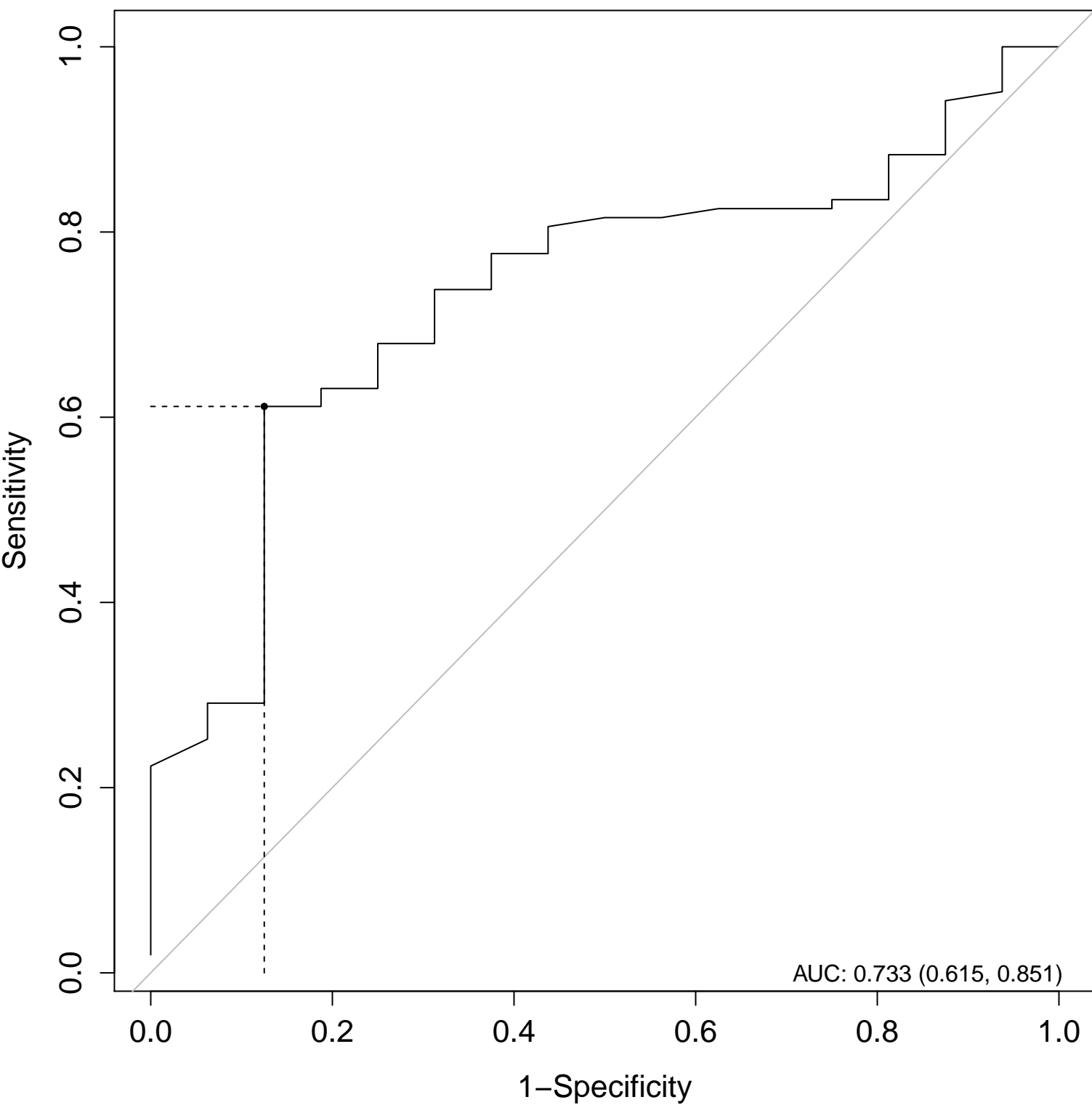

Supplement: S3 Appendix — (PDF) [file pone.0243447.s003.pdf]

ROC Curve. Criterion: MaxSp

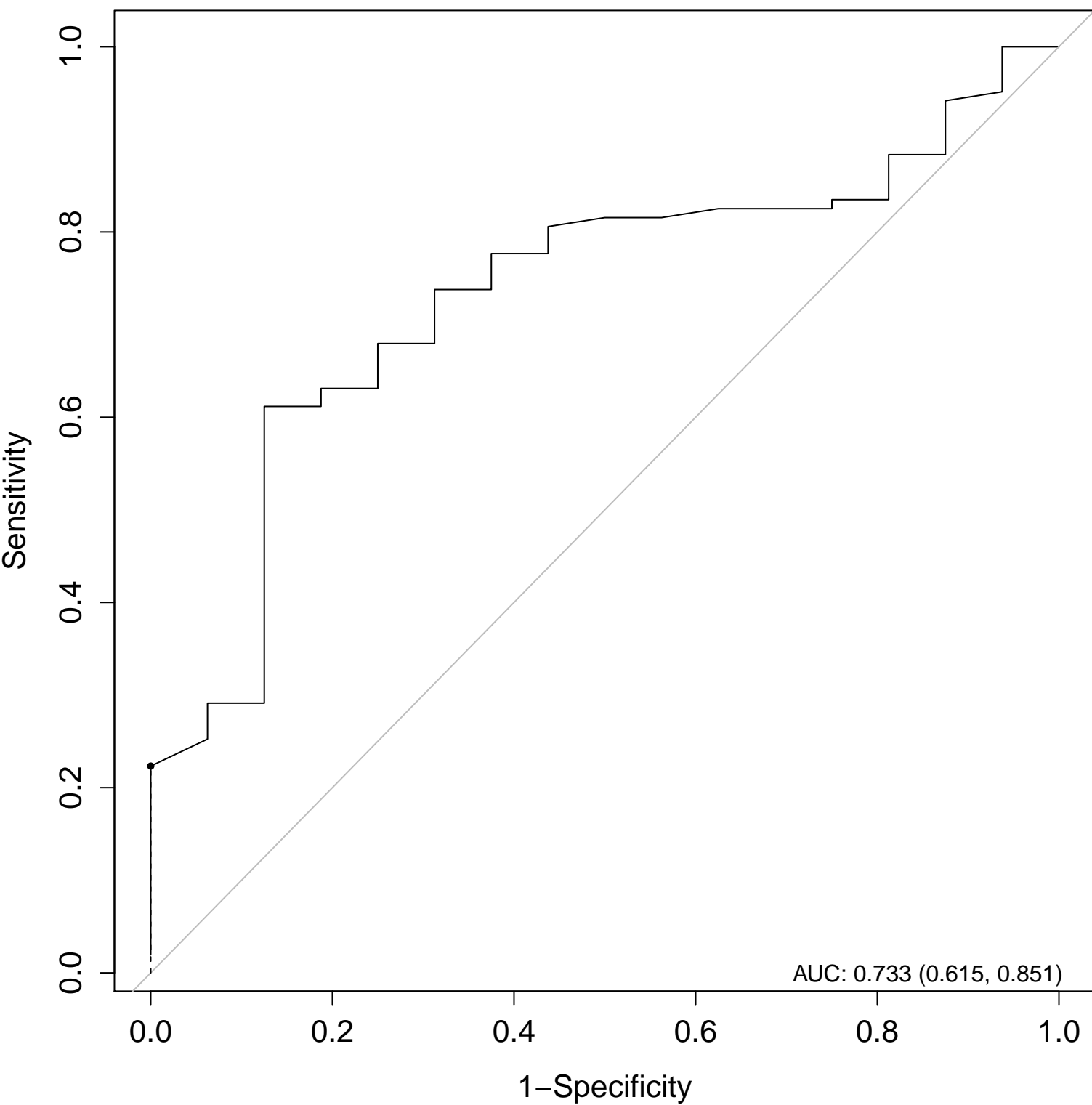

Supplement: S4 Appendix — (PDF) [file pone.0243447.s004.pdf]
