# Supplementary material for: Neutrophil-to-lymphocyte ratio predicts early mortality in females with metastatic triple-negative breast cancer
Source: PLoS One. 2020 Dec 7;15(12):e0243447. doi: 10.1371/journal.pone.0243447 (PMC7721150; doi:10.1371/journal.pone.0243447)
Supplement: S5 Appendix — (DOCX) [file pone.0243447.s005.docx]

S5 Appendix. Multivariate analysis of different cut-off values of the NLR

| Characteristics | Overall survival with NLR cut-off 3 | | | Overall survival with NLR cut-off 7 | | |
| --- | --- | --- | --- | --- | --- | --- |
|  | HR | 95% CI | P-value | HR | 95% CI | P-value |
| NLR |  |  |  |  |  |  |
| Low | ref | - | - | ref | - | - |
| High | 2.16 | 1.39-3.35 | 0.001 | 3.37 | 1.94-5.83 | <0.001 |
| Age | 1 | 0.98-1.01 | 0.695 | 0.99 | 0.98-1.01 | 0.355 |
| hCCI |  |  |  |  |  |  |
| Score 6 | ref | - | - | ref | - | - |
| Score ≥7 | 0.7 | 0.41-1.19 | 0.186 | 1.09 | 0.64-1.85 | 0.759 |
| Tumor size |  |  |  |  |  |  |
| T0-3 | ref | - | - | ref | - | - |
| T4 | 1.17 | 0.73-1.89 | 0.519 | 1.08 | 0.67-1.73 | 0.758 |
| Lymph node status |  |  |  |  |  |  |
| N0-1 | ref | - | - | ref | - | - |
| N2-3 | 1.09 | 0.71-1.67 | 0.708 | 1.07 | 0.7-1.64 | 0.758 |
| Places of metastases |  |  |  |  |  |  |
| 1 organ | ref | - | - | ref | - | - |
| ≥2 organs | 1.26 | 0.83-1.91 | 0.273 | 1.11 | 0.73-1.68 | 0.64 |
| Chemotherapy |  |  |  |  |  |  |
| No | ref | - | - | ref | - | - |
| Yes | 0.41 | 0.26-0.64 | <0.001 | 0.46 | 0.29-0.72 | 0.001 |
